# Exploring potential influencing factors of inadherence to specialist aftercare and long-term medication in patients with acromegaly

**DOI:** 10.1007/s11102-024-01400-4

**Published:** 2024-05-24

**Authors:** Sonja Siegel, Sabrina Giese, Jürgen Honegger, Anna Lena Friedel, Agnieszka Grzywotz, Karsten Henning Wrede, Ulrich Sure, Nicole Unger, Ilonka Kreitschmann-Andermahr

**Affiliations:** 1 Department of Neurosurgery and Spine Surgery, University of Duisburg-Essen, Hufelandstr. 55, 45147 Essen, Germany; 2 Department of Neurosurgery, University of Tübingen, Hoppe-Seyler-Str. 3, 72076 Tübingen, Germany; 3 Institute for Medical Education, University of Duisburg-Essen, Hufelandstr. 55, 45147 Essen, Germany; 4 Department of Endocrinology, Diabetes and Metabolism, University of Duisburg-Essen, Hufelandstr. 55, 45147 Essen, Germany

**Keywords:** Acromegaly, Adherence, Medication, Aftercare, WHO adherence model

## Abstract

**Purpose:**

To improve the understanding of adherence as one major factor of disease control in acromegaly patients, we systematically assessed patients’ motivations to adhere to advised follow-up schedules and recommended medication for acromegaly.

**Methods:**

Cross-sectional, postal questionnaire study on adult patients with acromegaly, operated upon a growth hormone producing pituitary adenoma more than 1 year ago in two tertiary treatment centers. We assessed demographic and clinical characteristics, disease status, adherence to acromegaly medication and/or aftercare, and the five dimensions defined by the World Health Organization influencing adherence. Wherever applicable, we included validated short scales. The answers of 63 patients (33 f, 30 m; mean age 56.1 y) were analyzed.

**Results:**

Patients with problems in adherence to aftercare had a significantly lower subjective symptomload than those adherent to aftercare (p = 0.026) and a lower perceived need for treatment (p = 0.045). Patients with adherence problems to medication had a higher subjective symptomload than those without (p = 0.056). They also tended to have shorter consultations, were significantly more often dissatisfied with the duration of their medical consultations (42% vs 4.8%, p = 0.019) and tended to find that their physician explained potential difficulties with adherence less well than patients without adherence problems (p = 0.089).

**Conclusions:**

To our knowledge, this is the first study which explored adherence to medication and aftercare in patients with acromegaly, taking into account potential influencing factors from all areas defined by the WHO model of adherence. Of the modifiable factors of adherence, patient–doctor relationship seemed to play a crucial role and could be one leverage point to improve adherence.

**Supplementary Information:**

The online version contains supplementary material available at 10.1007/s11102-024-01400-4.

## Introduction

Acromegaly is a rare, adult endocrine disorder caused by excessive growth hormone (GH) secretion after the closure of epiphyseal plates at puberty. In nearly all cases, it is caused by a GH secreting pituitary adenoma (for an overview see [1]). Persistent GH excess and that of its target hormone insulin-like growth factor I (IGF-I) leads to the insidious development of a characteristic acromegalic phenotype with acral overgrowth, macrognathia, and soft tissue swelling. It also results in a wide range of internal (i.e., metabolic, respiratory and cardiovascular), neurological and musculoskeletal comorbidities, which are oftentimes only partially improved or even persist unchanged with biochemical disease control (for an overview see [1]). Normalization of IGF-I and GH levels—with the concomitant reduction of excess mortality and morbidity—are the undisputed primary aims of acromegaly treatment. While surgical removal of the tumor is the therapy of first choice for acromegaly, many patients are in need of permanent medical treatment and/or other adjunctive therapies to achieve long-term disease control [1]. In others, the disease may relapse after initial postsurgical remission [2]. Thus, all patients with acromegaly necessitate regular visits to specialist (i.e. endocrinological and neurosurgical) aftercare and, furthermore, a large proportion needs to be adherent to medication in order to achieve normalization of disease activity.

According to the World Health Organization (WHO) adherence is defined as the extent to which a patient’s behavior corresponds with agreed recommendations from a health care provider [3]. The WHO model of adherence suggests that adherence to long-term therapies can be influenced by multiple factors from five dimensions including socioeconomic, condition-related, therapy-related, patient-related and health system-related factors. In acromegaly, these factors have not been investigated comprehensively, yet. Acromegaly patients from earlier studies state lack of information on the need for follow-up, difficulties to come to the visit, absence of symptoms or (unexplained) opposition to follow-up as reasons for inadherence [4, 5]. Patient interviews suggest that the patient-doctor interaction might play a role, too, as some patients felt unable to discuss adherence or problems with adherence with their doctor [6].

Against this background, we performed a cross-sectional questionnaire study on patients treated for acromegaly at the Departments of Neurosurgery and Spine Surgery and Endocrinology, Diabetology and Metabolism at the University Hospital Essen and the Department of Neurosurgery, University Hospital Tuebingen, Germany. We systematically assessed patients’ motivations to adhere to advised follow-up schedules and recommended medication for acromegaly and aimed to determine factors influencing adherence in this cohort.

## Methods

### Study design

We conducted a cross-sectional, multicenter, non-interventional, postal questionnaire study. The study followed an exploratory approach, using short scales to examine factors that may influence adherence. Factors for this study were selected based on the WHO model of adherence, which includes all five dimensions (i.e., socioeconomic, condition-related, therapy-related, patient-related, and health system-related factors). This approach serves to narrow down future research to those variables that have the potential to predict adherence in acromegaly.

### Study procedure

**Fig. 1:**
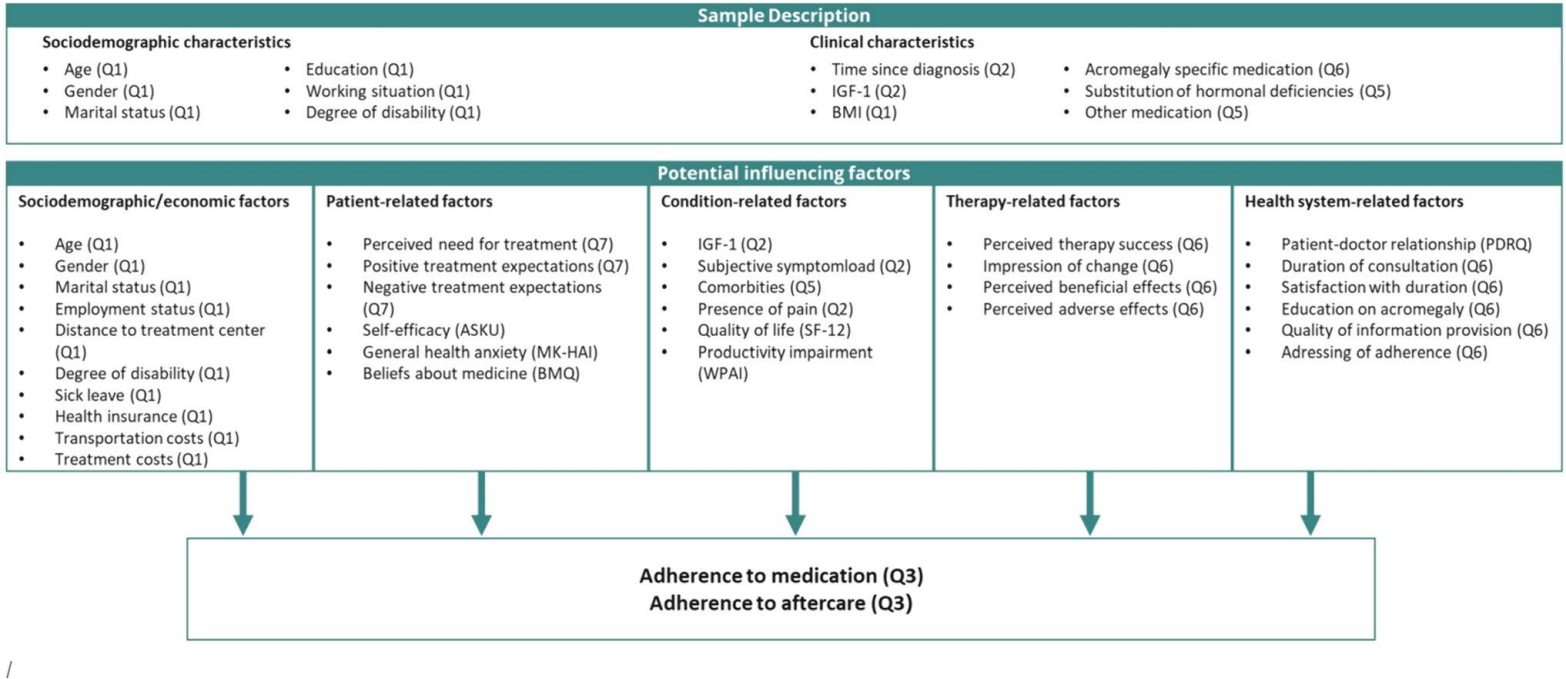
Overview of the investigated variables. Q1 to Q7: self-developed questionnaires, ASKU: General Self-efficacy Scale, MK-HAI: German Modified Health Anxiety Inventory, SF-12: 2-item Short-Form Health Survey, PDRQ: Patient–Doctor Relationship Questionnaire, BMQ: Beliefs about medicine questionnaire, Work Productivity and Activity Impairment questionnaire, BMI: body mass index

We contacted adult patients with acromegaly more than 1 year after treatment initiation and either currently in aftercare in the participating clinics or previously in treatment but lost to follow-up. The study had been approved by the ethics boards of the University of Essen and the University of Tübingen. All eligible patients received by mail the same questionnaire package covering the aspects displayed in Fig. 1.

### Definition of adherence

For this study, we compared patients with and without problems in adherence. Patients were defined as adherent to medication, if they stated in the Q3-questionnaire that they did not forget their acromegaly-specific medication intake within the last four weeks, did not forget medications at home, did not skip a dose or reduce a dose on purpose. Patients were defined as adherent to aftercare, if they went to endocrinological or neurosurgical aftercare at least once a year.

### Sample description

Sixty-eight patients (41 from the center in Essen, 27 from Tübingen) returned the filled-out questionnaires. Of these, 5 were excluded from the analysis due to missing consent forms or incomplete answers. Therefore, the surveys of 63 patients could be analyzed (33 women and 30 men). The mean age of the patients included in the analysis was 56.1 (SD 14.2) years. At enrolment, the mean time since the diagnosis of acromegaly was 11.8 (SD 8.3) years. Sixty-one patients (96.8%) had undergone neurosurgery, 8 (12.7%) had received radiation therapy. At the time of the study, 43 patients (68.8%) were on medication. Fourty-one (85.4%) of the patients had normal IGF-1 levels. Patients were on average overweight (mean BMI 28.8, SD 5.3) and one fifth reported to take analgesics, either on a regular basis or on demand.

Sociodemographic and clinical characteristics of the study sample are detailed in Table 1.

**Table 1:** Sociodemographic an clinical characteristics of the study sample (N = 61)

Sociodemographic characteristics			Clinical characteristics		
Variable	M (SD)	n (%)	Variable	M (SD)	n (%)
Age	56.1 (14.2)		Time since diagnosis	11.8 (8.3)	
Gender			Normalized IGF-1		41 (85.4)
Male		30 (47.6)	BMI	28.8 (5.3)	
Female		33 (52.4)	Acromegaly specific medication		
Marital status			Somatostatin analogues		23 (36.5)
Single		14 (22.6)	Dopamine agonists		3 (4.8)
Steady relationship/married		41 (66.1)	GH-receptor antagonists		14 (22.2)
Divorced		2 (3.2)	Substitution of hormonal deficiencies		
Widowed		5 (8.1)	Substituted gonadotropic deficiency		2 (3.2)
Education			Substituted thyrotropic deficiency		14 (22.2)
Lower secondary school diploma		19 (31.1)	Substituted corticotropic deficiency		4 (6.3)
Higher secondary school diploma		17 (27.9)	Substituted deficiency of the posterior pituitary		4 (6.3)
Specialized high school diploma		7 (11.5)	Other medication		
High school diploma		18 (29.5)	Antidepressants		4 (6.3)
University degree		18 (29.5)	Antihypertensives		27 (42.9)
Working situation			Antidiabetics		7 (11.1)
Full time		25 (41.7)	Analgesics		13 (20,6)
Part time		7 (11.7)	Anticoagulants		5 (7.9)
Disability pension		4 (6.7)	Lipid-lowering agents		6 (9.5)
Retired/ not working for other reasons		24 (38.1)	Vitamin D3		11 (17.5)
Officially recognized degree of disability		30 (49.2)			

BMI: body mass index

## Materials and methods

We developed a set of questionnaires to measure adherence and potential influencing factors as stated by the WHO [3]. Wherever applicable we included validated short scales as outlined below. Since the study aimed to include many different factors without placing excessive time demands on the participants, no longer questionnaires were included.

### Self-developed questionnaires

Overall, seven self-developed questionnaires were used in this study covering sociodemographic questions (Q1), current disease status (Q2), adherence to acromegaly-specific medication (Q3), comorbidities (Q4), course of the therapy (Q5), doctor-patient-communication (Q6) and perceived therapy needs (Q7). The questionnaires consisted of yes/no-items, multiple choice questions, free-text questions and 5-point Likert-scale items (0 to 4). When several Likert-scaled items referred to the same topic (e.g. positive treatment expectations) a mean score of the respective items was calculated. The full questionnaires can be found in the supplemental material, scales for which a mean score was calculated are marked.

### 12-item Short-Form Health Survey, SF-12

The SF-12 [7] is an abridged version of the established 36-item Short-Form Health Survey (SF-36), a generic questionnaire to measure health-related quality of life (QoL). It consists of a total of 12 items and allows to differentiate between physical and mental QoL by calculation of two independent scores: the Physical Component Summary (SF-12 PCS) score, and the Mental Component Summary (SF-12 MCS) score. For comparison with the age- and sex-matched reference values of the German normative population, the raw values were transformed into standardized scores [8]. The transformed summary scores have a mean of 50 and SD of 10. Higher scores indicate a better QoL.

### Patient–Doctor Relationship Questionnaire 9, PDRQ-9

The PDRQ-9 [9] is a validated 9-item questionnaire to assess the alliance between doctor and patient. Patients indicate on a five-point Likert scale ranging from 1 to 5 how appropriate they find statements concerning their treating physician. A mean score can be calculated from the results. Higher values are interpreted as a better patient–doctor relationship.

### General Self-efficacy Scale, ASKU

The ASKU (German: Allgemeine Selbstwirksamkeits- Kurzskala) [10] is a 3-item short scale to measure the patients’ expectation to be able to overcome day-to-day obstacles. Answers are given on a five-point Likert Scale ranging from 1 to 5, higher values signify a better self-efficacy. German reference values for different age groups and genders are available.

### German Modified Health Anxiety Inventory, MK-HAI

The MK (German: modifizierte Kurzform)-HAI [11] is a validated short scale to measure patients’ tendency towards health-related concerns. It is a German abbreviated form of the Health Anxiety Questionnaire [12]. The questionnaire consists of 14 statements concerning patients’ health anxiety in the last 6 months. Patients indicate how much they agree with these statements on a five-point Likert Scale ranging from 0 to 4. The results are added up to a sum score with higher values meaning worse health anxiety.

### Beliefs about medicines questionnaire, BMQ

The BMQ [13] investigates cognitive and emotional representations of medication. In the present study, the German translation of the BMQ by U. Opitz was used [14]. It comprises 4 factors, assessing (1) beliefs that medicines are harmful, addictive poisons which should not be taken continuously (General Harm), (2) beliefs that medicines are overprescribed by doctors (General Overuse) (3) beliefs about the necessity of prescribed medication (Specific Necessity) and (4) concerns about prescribed medication based on beliefs about the danger of dependency, long-term toxicity and the disruptive effects of medication (Specific Concerns) (Horne, 1999). For each of the scales the average score can be calculated. To assess the balance between perceived benefits and costs of the prescribed medication, the difference between Specific Necessity and Specific Concerns can be calculated (Horne, 1999b). The resulting score ranges from − 20 to 20, with negative values indicating that perceived concerns exceed benefits and positive scores indicating that perceived benefits exceed concerns.

### Work productivity and activity impairment questionnaire, WPAI

The WPAI [15] consists of 6 items measuring the amount of time in which patients activities were impaired due to health problems. It asks for work hours missed, affected productivity during work and during other activities. The overall impairment in work and other activities can be calculated. For patients, who are not employed, only activity impairment is assessed. The results are given in percent of time with impairment.

### Data analysis

SPSS 29.0 (Statistical Package of the Social Sciences, IBM, Armonk/USA) for Windows was used to analyze data. Interval scaled data were described as mean (M) and standard deviations (SD) for the total study group and as median and range for subsample analyses to avoid distortions in smaller groups. Categorical data are given as valid percent. All questionnaire scores were compared by medical adherence and adherence to aftercare. For the group comparisons, Mann–Whitney U tests for unpaired variables were used to account for unequal group sizes and non-normal data. Categorical data were compared using Chi Square tests and Fisher’s exact tests. Since this research approach was explorative, we decided against alpha error corrections in order to decrease the risk of falsely dismissing relevant factors (beta error). An alpha-level of p = 0.05 was considered significant, but nearly significant results were also included in the report. Only those patients, who received medical therapy were included in the analyses regarding adherence to medication, resulting in a sample size of N = 43 for these analyses. All other analyses were calculated with the full sample of N = 61. Missing values were regarded as missing at random and were deleted casewise from the analysis.

## Results

### Adherence

With a mean adherence score (Q3) 16.8 ± 1.4 (out of 18) the average adherence was high in the sample. 22 patients (51.2%^1^) stated to have any problems with adherence (i.e. forgot medications in the last 4 weeks, forgot medications at home, skipped a dose or reduced a dose on purpose). Fifty-one patients (81.0%) were adherent to aftercare, i.e. went to endocrinological or neurosurgical aftercare at least once a year. All patients either fully (n = 63, 87.8%) or rather agreed (n = 5, 12.2%) to take their medication because of the advice of their treating physician. Twenty-two of the patients (56.4%) stated to take their medication to improve their physical capacity and 15 (38.5%) took their medication to improve their mental health. Pain reduction was the reason for adherence in 14 (36.9%) of the patients.

### Sociodemographic/economic factors

Patients with any problems in adherence to medication were almost significantly younger than patients without problems in adherence (p = 0.053). Patients with problems in adherence to aftercare had a significantly longer distance to their treatment center than patients’ adherent to aftercare (p = 0.048). There were no other group differences with regard to gender, marital status, education level, employment status, percentage of patients with a degree of disability or on sick leave, percentage of patients with a private health insurance, transportation costs per appointment and costs of medication. Table 2 displays all significant group differences.

**Table 2:** Group differences between patients with and without problems in adherence to medication (N = 43) and to aftercare (N = 61)

Related factors	Adherence to medication					Adherence to aftercare				
	No problems with adherence		Any problems with adherence		p	Adherent		Not adherent		p
	Median (Range)	n (%)	Median (Range)	n (%)		Median (Range)	n (%)	Median (Range)	n (%)	
Sociodemographic factors										
Age	63 (24 to 81)		54 (26 to 79)		0.053					
Distance to treatment center						40.0 (3 to 85)		60.0 (50 to130)		0.048
Patient-related factors										
BMQ-Difference	8.5 (3–19)		3 (− 9 to 15)		0.004	7.0 (− 9 to 19)		3.0 (− 9 to 11)		0.038
Perceived need for treatment						3.0 (0 to 4)		1.0 (0 to 3)		0.045
Condition-related factors										
Subjective symptomload	0.9 (0.3 to 1.9)		1.4 (0.1 to 2.4)		0.056	0.9 (0.1 to 2.4)		0.6 (0.0 to 1.5)		0.026
Mental QoL (SF-12)	54.2 (29.2 to 65.3)		43.7 (23.2 to 61.2)		0.025					
Health-system-related factors										
Duration of consultations										
Less than 5 min		0 (0%)		1 (4.5%)	0.106					
5 to 10 min		5 (23.8%)		9 (40.9%)						
10 to 20 min		12 (57.1%)		12 (54.5%)						
More than 20 min		4 (19.0%)		0 (0.0%)						
Satisfaction (consultation duration)										
Exactly right		20 (95.2%)		13 (59.1%)	0.019					
A little too short		1 (4.8%)		7 (31.8%)						
Much too short		0 (0%)		2 (9.1%)						
Addressing problems with adherence	3.00 (2 to 4)		3.0 (0 to 4)		0.089	3.0 (0 to 4)		2.0 (2 to 3)		0.077

Displayed are only those factors, which were significantly or nearly significantly related to adherence to medication (i.e. patients did not forget their acromegaly-specific medication intake within the last four weeks, did not forget medications at home, did not skip a dose or reduce a dose on purpose) and adherence to aftercare (i.e. patients visited endocrinological or neurosurgical aftercare at least once a year) as measured in Q3

BMQ: Beliefs about medicine questionnaire, QoL: quality of life, SF-12: 12-item Short-Form Health Survey

### Patient-related factors

Five of the patients (8.6%) perceived no *need for treatment (Q7)*, 9 (15.5%) a low, 16 (27.6%) moderate, 21 (36.2%) high and 7 (12.1%) very high need for treatment. The most frequently named *positive treatment expectations* (Q7) were for the treatment to alleviate physical complaints (n = 47, 83.9%), to improve performance in everyday life (n = 45, 80.3%) and to improve mental health (n = 39, 69.6%). Eighteen of the patients (34.6%) expected a quick therapeutic success, 20 (41.6%) expected the medication to prevent the need for pituitary surgery. A third of the patients stated, that they expected their treatment to result in a reduced need for medication (n = 17, 34.0%) or doctor’s appointments (n = 18, 31.5%). Patients reported less *negative treatment expectations (Q7)* than positive treatment expectations. Most frequently, patients worried that the treatment would not lead to the improvement they hoped for (n = 20, 37.7%), that it would have unpleasant side effects (n = 19, 36.5%) or that it would not be effective (n = 14, 26.0%). Ten of the patients (18.9%) worried that the treatment would dominate their everyday life and 5 (9.6%) that their symptoms would worsen with treatment. The *self-efficacy (ASKU)* score was on average 4.0 (SD 0.9). This corresponds to a medium self-efficacy according to the reference values of the inventory (the general population mean is 4.0 ± 0.74 [10]. The average *general health anxiety (MK-HAI)* was 35.4 (SD 12.6) from a possible maximum of 56, signifying health anxiety above the population average (population mean for men 12.48 ± 9.92 and for women 14.43 ± 10.71 [11]). With regard to their *beliefs about medicine (BMQ)*, 46 of the patients (83.6%) reached a Specific Necessity Score above midpoint, indicating a strong belief in the need for the medication. Fourteen (25.9%) had strong Specific Concerns about potential negative effects of their medication. The average Necessity-Concerns Difference was 6.5 (SD 6.4), indicating that most patients judged the benefit of their medication to exceed their concerns.

#### Group differences

Patients stating any problems with *adherence to medication (Q3)* did not differ significantly from patients stating no problems with adherence with regard to their *perceived need for treatment (Q7)* or their *positive and negative treatment expectations (Q7)*. Specific concerns in the questionnaire on *beliefs about medicine (BMQ)* outweighed the beliefs of the necessity of the medication more often in patients stating any problem with adherence to medication (p = 0.004) and in patients who stated problems with *adherence to aftercare (Q3)* (p = 0.038). Patients with problems in adherence to aftercare also had a significantly lower *perceived need for treatment (Q7)* than patients adherent to aftercare (p = 0.045).

### Condition-related factors

**Fig. 2:**
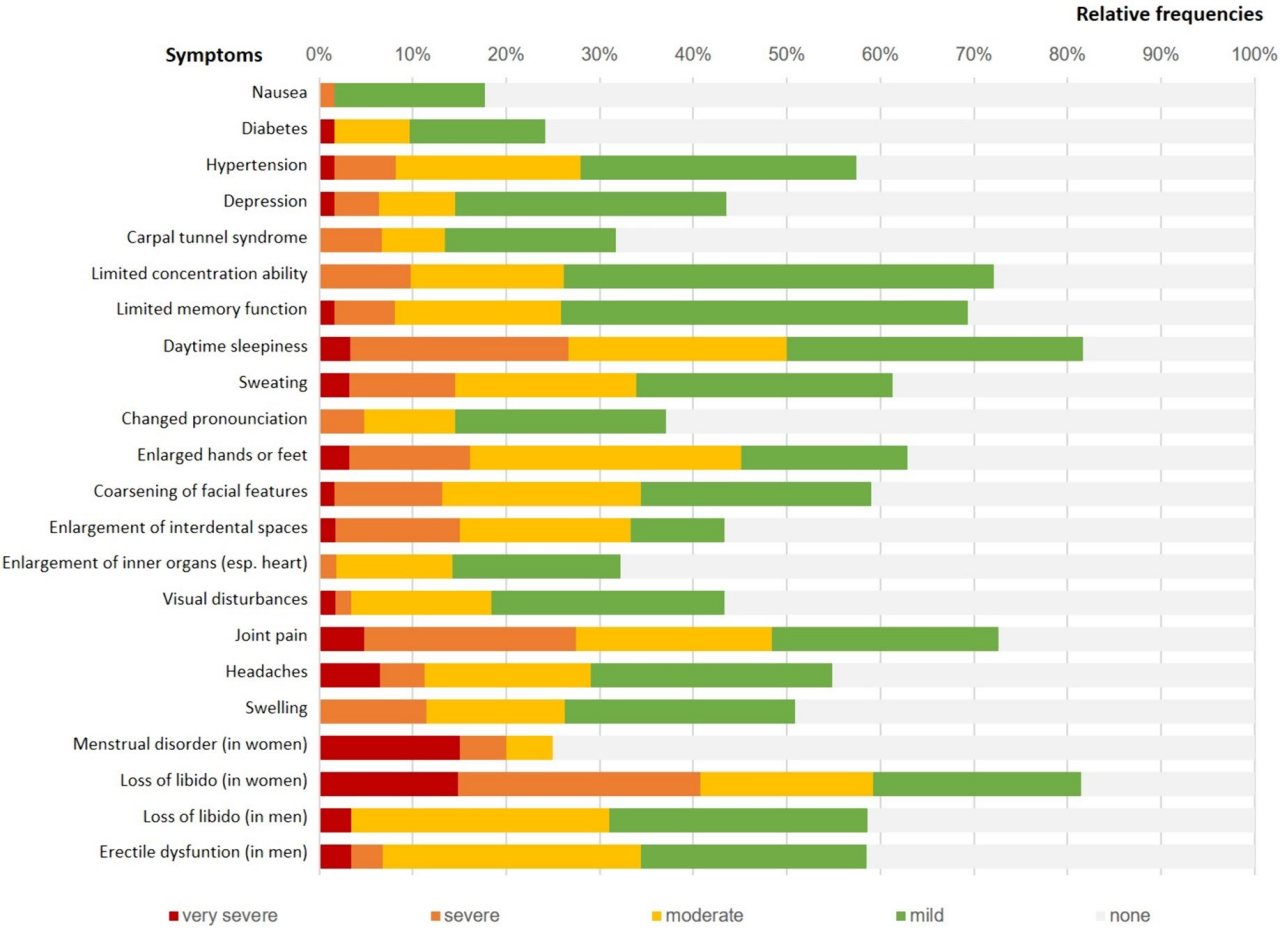
Subjective symptomload as reported in Q2 (N = 62)

Asked for their *subjective symptomload (Q2)*, patients stated that they suffered the most from daytime sleepiness (n = 16, 26.6% severe/very severe), joint pain (n = 17, 27.4% severe/very severe) and loss of libido in women (n = 11, 40.7% severe/very severe; cf. Fig. 2).

**Fig. 3:**
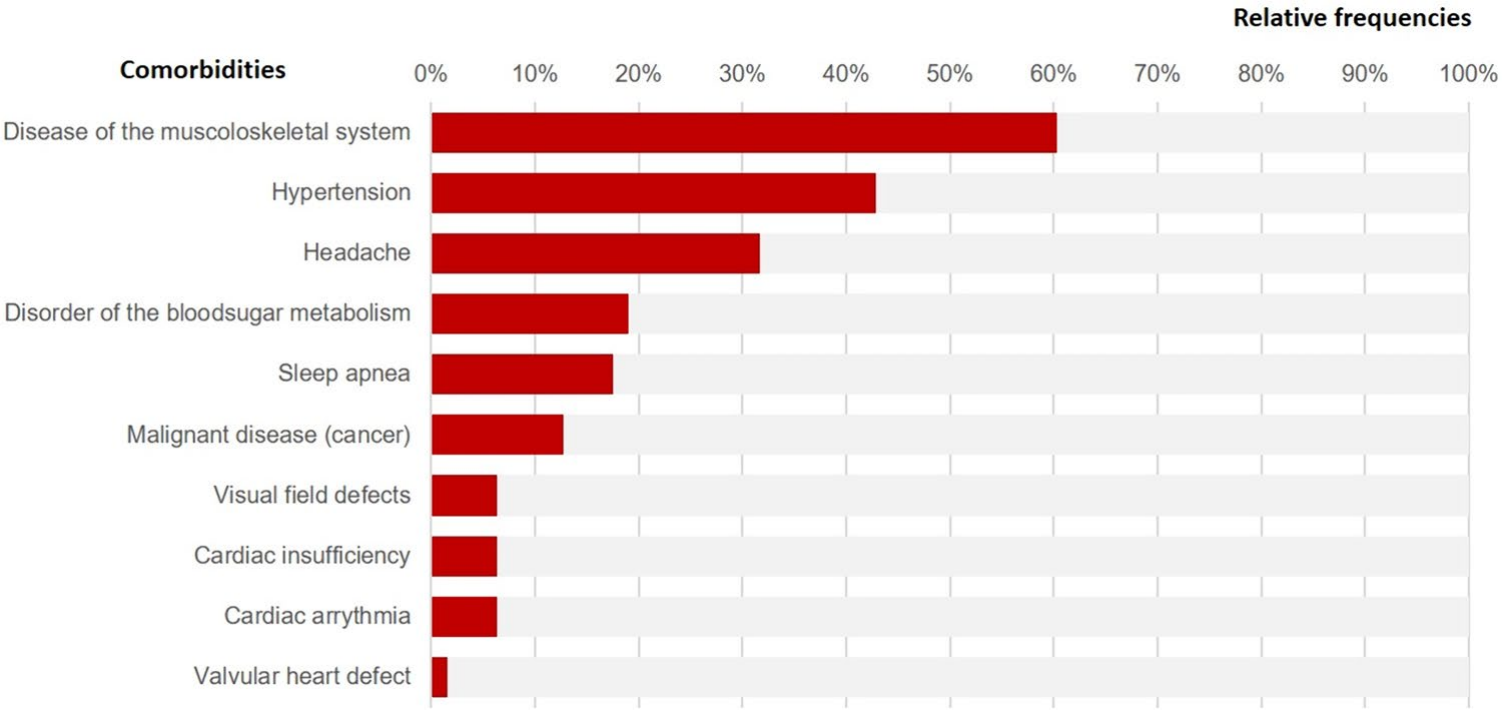
Relative frequencies of comorbidities (N = 63)

Twelve (19%) of the patients had no *comorbidities (Q5)* and 9 (14.3%) had only common comorbidities (hypertension, headache, sleep apnea). Twenty-seven (42.9%) had at least one severe comorbidity (muscosceletal diseases, visual field defects, diabetes) and 15 (23.8%) had at least one potentially life-threatening disease (cardiac disease, malignant diseases)**.** The frequencies of the individual comorbidities are displayed in Fig. 3.

Nine of the patients (15.3%) reported a physical *QoL (SF-12)* 1 SD below average and 7 (11.9%) a physical QoL 2 SD below average. Six patients (10.2%) reported a mental QoL 1 SD below average and 10 (16.9%) 2 SD below average. The overall *activity impairment (WPAI)* reported by the patients in the WPAI was 31.2% (SD 28.4%). Those patients who worked full- or part-time reported an average work impairment of 20% (SD 21.0%). 4 patients (14% of the working patients) stated to have missed work time due to health issues.

Group Differences. Patients stating any problems with adherence to medication (Q3) had an almost significantly higher *subjective symptomload (Q2)* than patients stating no problems with adherence (p = 0.056). *Mental QoL (SF-12*) was significantly worse in patients who stated any problems with adherence than patients that did not (p = 0.025). Patients with problems in adherence to aftercare (Q3) had a significantly lower *subjective symptomload (Q2)* than patients adherent to aftercare (p = 0.026). The percentage of patients with normalized *IGF-1 levels (Q2)* did not differ between the groups. There was no difference between the groups with regard to the level of *comorbidities (Q5)* and *presence of pain (Q2)*.

### Therapy-related factors

*Perceived therapy success –(Q6)* was high in most patients**.** Twenty patients (35.1%) perceived their therapy as very successful, 24 (42.1%) as successful, and 11 (19.3%) as moderately successful. Only 2 patients (3.5%) rated their therapy as little successful or not successful at all. Regarding their *impression of change (Q6*), 9 patients (15.8%) reported that their physical well-being had much improved since the beginning of their therapy and 22 (38.6%) that it had a little improved. 17 (29.8%) had the impression that their physical well-being was unchanged and 9 (15.8%) that it had deteriorated. With regard to mental well-being, 7 patients (12.3%) reported that it had much improved and 19 (33.3%) that it had a little improved since the start of the therapy. Twenty-four patients (42.1%) found their mental well-being to be unchanged and 7 (12.3%) found that it had worsened. The most frequent *perceived beneficial treatment effect (Q6)*, reported by 45 of the patients (84.9%) was that they felt they could be more open about their disease now. Thirty-three of the patients (62.2%) agreed, that they could handle their daily life better again. Fourty-one patients (77.4%) stated that they now understood the changes acromegaly causes and 34 (64.4%) that they could accept them. *Perceived adverse effects (Q6)* were less frequently reported than positive effects. Thirteen of the patients (28.2%) reported to suffer from negative side effects and 8 (17.7%) to be burdened by the daily medication intake. Seven patients (13.4%) felt stressed by the medical appointments and 3 (5.7%) stated that therapy took up a lot of space in their life.

#### Group differences

There were no significant group differences with regard to therapy-related factors.

### Health system-related factors

**Fig. 4:**
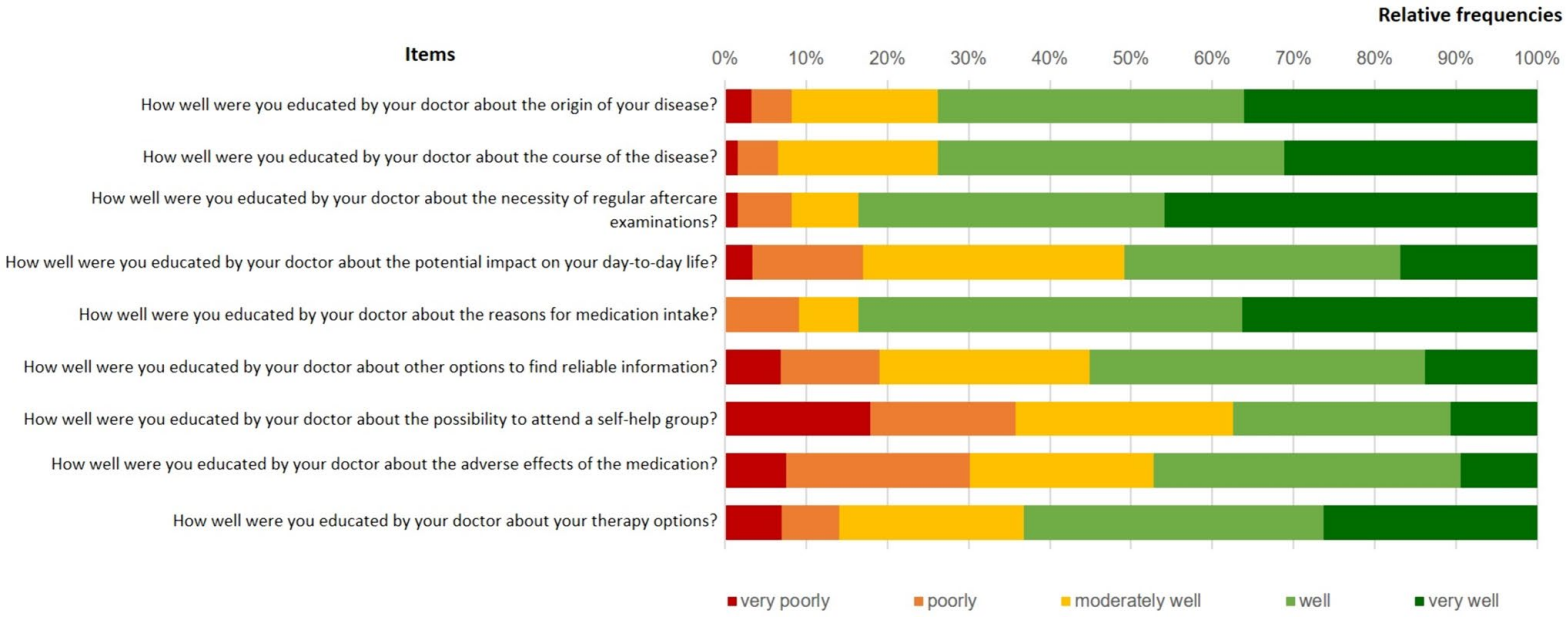
Education on acromegaly as reported in Q6 (N = 61)

The mean *patient-doctor-relationship (PDQR-9)* score was 35.1. This corresponds to a t-value of 36, which points to a below-average patient-doctor-relationship. Patients were overall well satisfied with the *education on acromegaly (Q6)* they received during their therapy. However, more than one-third of all patients (n = 20, 35.7%) felt to be informed poorly or very poorly on the possibility to attend a self-help group. Education on potential adverse effects of medication was rated as poor or very poor by 16 patients (30.2%) and education on the effects of acromegaly on day-to-day life was also rated poor or very poor by 10 of the patients (16.9%) in the study sample (cf. Fig. 4).

**Fig. 5:**
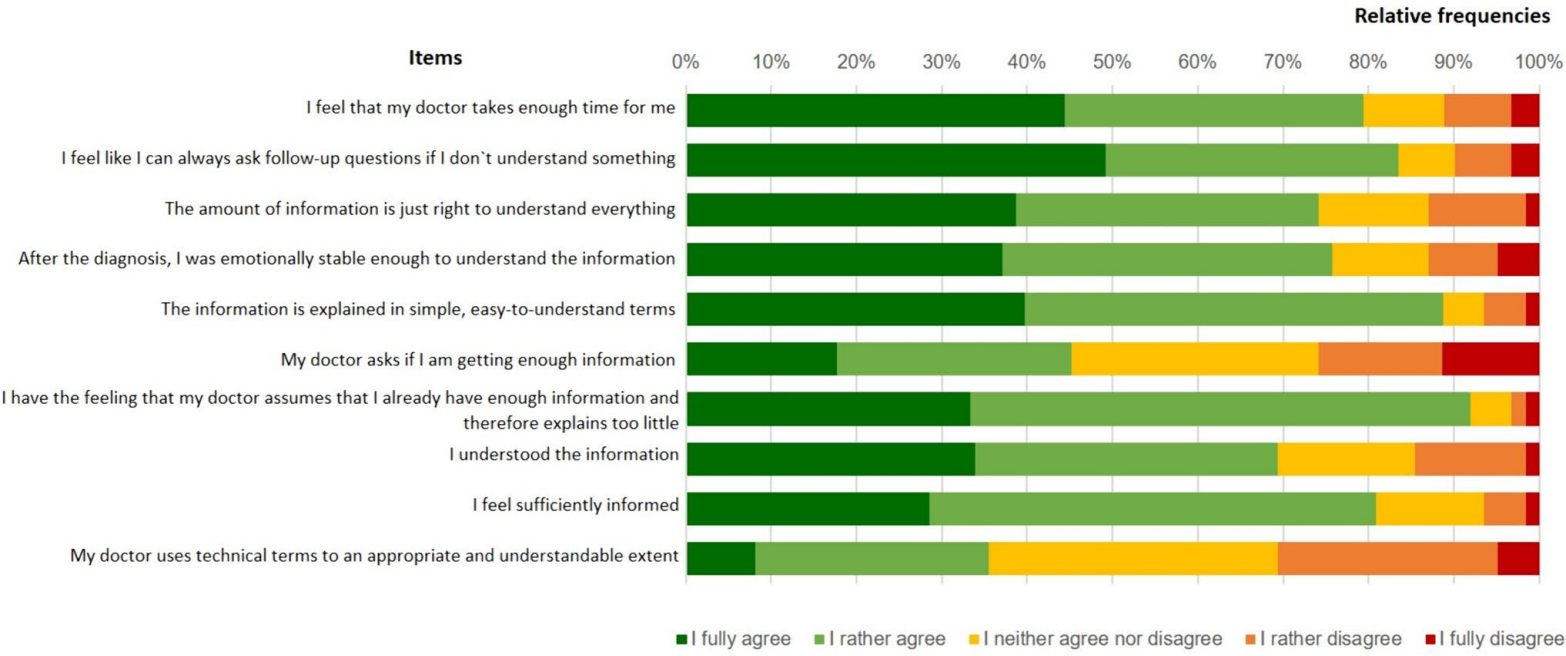
Quality of information provision as reported in Q6 (N = 63)

Most patients were satisfied with the *quality of information provision (Q6).* Fifty patients (79.3%) agreed, that their doctor took enough time for them and 56 (88.9%) that their doctor used easy-to-understand terms. But only 28 patients (45.1%) reported, that their doctor asked them, whether the amount of information received was sufficient (cf. Figure 5).

#### Group differences

Patients stating any problems with *adherence to medication (Q3)* tended to have a shorter duration of consultations and were significantly more often dissatisfied with the *duration of their medical consultations (Q6)* than patients stating no problems with adherence (42% vs 4.8%, p = 0.019). Patients who reported problems with adherence to medication had a nearly significant tendency to find that their physician *addressed potential problems with adherence (Q6)* less well than patients without problems with adherence (p = 0.089). There was no difference with regard to *education on acromegaly (Q6)* and *information provision (Q6)* between the groups. *Adherence to aftercare (Q3)* was unrelated to health system-related factors.

## Discussion

To our knowledge, this is the first study, which explored influencing factors of adherence to medication and aftercare in a large cohort of patients with acromegaly. In this exploratory approach, we investigated potential influencing factors based on a theoretical model of adherence with the aim to identify the most relevant factors for patients with acromegaly. We found different factors to be important for adherence to medication compared to adherence to aftercare. Overall, our data suggest, that those patients with a self-assessed higher disease severity struggle more with adherence to medication. They were younger, had a higher subjective symptomload and worse mental QoL. On the other hand, patients with lesser symptoms had more problems with adherence to aftercare. These patients had a lower subjective symptomload and a lower perceived need for treatment.

### Adherence to acromegaly-specific medication

Adherence to acromegaly-specific medication in the study group was very good, with approximately one half of the investigated patients reporting perfect adherence to their prescribed medication, whereas the other half reported to skip or reduce a dose at least now and then. No patient reported not to take their prescribed mediation at all.

The main reason for taking acromegaly medication was the advice of their treating physician, followed by a self-perceived improvement of physical and mental capacity. Interestingly, almost 40% of the patients in our sample stated to take their acromegaly medication because they felt that it reduced pain. Unfortunately, our data do not allow to differentiate whether this is connected to a described effect of somatostatin analogs on reversibility of joint thickening in acromegalic arthropathy [16, 17] or other factors. However, it is an interesting finding, worth to be elucidated further in future studies.

Of all *sociodemographic/economic factors* potentially impacting on adherence and investigated in the present study, we found only age to be of relevance for medication adherence in the direction that patients in this study sample stating problems with adherence were younger than those without. The same finding also emerged in a previous study by our group on adherence to GH replacement therapy [18]. Younger age as an influencing factor for poorer adherence has also been uncovered in other population-based studies, for example from Germany and Sweden [19, 20] and has been discussed to be related to perceived side effects of medication [3, 19]. It has also been found to be a relevant factor for treatment persistence in patients with acromegaly [21].

*Condition-related factors* also played a crucial role in adherence to medication. Patients with any problems of adherence to acromegaly medication in this study reported a significantly poorer mental QoL and a higher acromegaly symptomload. Similarly, a recent Bulgarian study found inadherence to acromegaly medication to be related to impaired QoL in the SF-36 [22]. These findings do also not come as a surprise. Across studies, poor mental QoL is highly associated with depressive symptomatology [23]. Depression itself, which is known to be related to negative illness beliefs, helplessness and lack of perceived illness control [24] is a known barrier to adherence [25]. We were unable to determine from our data, whether the higher symptomload, that also accompanied adherence problems in our study, is an expression of depressive symptomatology or a consequence of adherence problems. Probably, these factors are mutually dependent. Patients with a higher symptomload and a poorer QoL find it more difficult to take their medication, potentially because of a higher prevalence of depression and depressive coping strategies in this group. Lack of adherence in turn reduces the likelihood of successful treatment. This relationship may contribute to the fact that the QoL of many patients with acromegaly remains permanently reduced [26, 27]. Supporting these patients in acquiring better coping strategies and achieving better adherence might be a valuable leverage point to improve their psychological well-being. Interestingly, IGF-1-normalization was not related to adherence, perhaps indicating that the perceived disease severity according to symptomload and subjective QoL may be more relevant to patient motivation than hormonal status.

With regard to *health system related factors*, we identified the length of the medical consultation to be impacting on adherence to medication in patients with acromegaly. The patients with adherence problems tended to have a shorter duration of consultations and were significantly more often dissatisfied with the duration of their medical consultations than patients stating no problems with adherence. They found that physician did not address potential adherence problems well, significantly more often, then patients without problems in adherence. The importance of a good patient-provider relationship for adherence has been consistently shown in previous studies in patients with chronic health conditions (for an overview see [28, 29]). This finding underlines the importance of the communication skills of the treating physician for the success of medical treatment of acromegaly. This notion is supported by patient interview studies in which some acromegaly patients felt unable to discuss adherence problems with their doctor [6].

### Adherence to aftercare

Adherence to aftercare in our study was also excellent, with over 80% of all patients reporting to see their endocrinologist and/or neurosurgeon at least once a year. Only few of all constructs investigated impacted on adherence to aftercare: In contrast to patients who had problems with medication adherence, patients with problems in adherence to aftercare had a lower symptomload and perceived need for treatment. This is in line with findings from Kasuki, who reported absence of symptoms to be one of the main reasons for loss to follow-up in patients with acromegaly in a pilot study across three treatment centers [5].

Interestingly, none of the patients who did not visit aftercare appointments regularly lived closer than 50 km away from the treatment center. These results suggest that the regular attendance of aftercare seems to be dependent on the patients understanding of its necessity and their willingness to take on long travel times to the treatment center. The effect of increased distance to the treatment center has been shown to be associated with an increase in likelihood of non-adherence to treatment or follow-up plans and—in some illnesses, even survival—in other diseases [30–32] in pediatric and adult populations. Our findings serve as arguments for increasing necessity beliefs about aftercare in acromegaly patients as well as the expansion of telemedicine services, especially in those patients with a milder course of the disease. Experience with telemedicine during the Covid-19 pandemic suggests, that adherence rates in patients with acromegaly can be significantly improved if the treating physicians stays in contact via online visits [33].

The only factor influencing both adherence to medication and adherence to aftercare, was a higher degree of concerns about medication than the belief in their necessity. This result is in line with a meta-analytic review of the necessity-concerns framework published in 2013 [34] in which across studies, higher adherence was associated with stronger perceptions of necessity of treatment and fewer concerns about treatment, independently of study size, country in which the research was conducted and the type of adherence measure used. In the context of our study, it can be seen as an important reminder that the patients’ concerns should be addressed and resolved during medical consultations to improve adherence in patients with acromegaly.

### Strengths and limitations

The major strengths of our study are a novel approach to measure adherence and influencing factors, combining self-designed questionnaires with standardized ones, and taking into account all the five dimensions on adherence defined by the WHO. Despite the considerable size of the questionnaire package, we managed to achieve a sufficiently large sample to obtain statistically meaningful results. The scientific approach to construct a study design based on a theoretical model to then eliminate irrelevant factors in an explorative study, allows a systematic investigation of adherence in acromegaly.

To address questions for which standardized questionnaires are not available, this study had to rely largely on self-developed questionnaires that have not been used before and for which normal values are not available. It is therefore a limitation of this study that our results cannot be compared to a healthy population. Also, the greater time efficiency of the validated short scales allowed us to examine more different factors in the same sample, but does not allow for the same in-depth analysis as their respective longforms. The large number of items was necessary for the exploratory research approach, but may also have led to a higher number of missing values, which we have dealt with by reporting valid percentages.

Therefore, it has to be kept in mind that the factors identified in this study are in need of further confirmation. We purposefully decided to include factors that were only nearly significant and to refrain from corrections for alpha errors. This limits the certainty with which we can identify influencing factors in this study, but serves the main purpose of this study to narrow down future research to those variables that have the potential to predict adherence. It is important at this explorative stage of the scientific understanding of adherence, to not falsely exclude factors, which would then not be further investigated. We suggest, to conduct prospective studies including all factors we found to be associated with adherence in this study, to further prove their actual influence.

An unforeseen limitation of this study was, that due to a change of date protection legislation, which went into effect after the finalization of the study plan, it was no longer possible to trace back former patients of the participating institutions to enquire about their present aftercare status and medication adherence. This limited the information we were able to accrue on patients lost to follow-up. We also have to acknowledge a potential response bias in that we cannot rule out that predominantly highly motivated and adherent patients participated in this study, who might not constitute a representative sample of the general group of patients with acromegaly. On the other hand, self-reports are susceptible to error and filling in in terms of social desirability, thus potentially overestimating the degree of adherence in our patients. However, this is a problem shared by all studies relying on self-reporting measures and cannot be avoided in patient-reported outcome research.

## Summary and outlook

In conclusion, we found adherence to medication and adherence to aftercare to be independent concepts, which are associated with different patient characteristics. They should be investigated separately in future studies and addressed individually with the patients. The adherence model of the WHO proved a valuable starting point for the identification of relevant influencing factors. While the concept is broad and designed to suit many different chronic diseases, it well encompasses the specific problems of adherence in acromegaly as a rare disease. For adherence to medication, we found the patient–doctor relationship to be of crucial importance and a potential leverage point to improve adherence. For the adherence to aftercare, the perception of treatment necessity and easy availability of specialist care were central. The high treatment motivation we observed in our sample was in a huge part driven by a hope for pain alleviation. The burden of pain for patients with acromegaly might be underestimated and clearly needs to be investigated further.

### Supplementary Information

Below is the link to the electronic supplementary material.

Supplementary file1 (PDF 1365 KB)

## Data Availability

No datasets were generated or analysed during the current study.

## Footnotes

1 The result section reports valid percent based on only those patients who answered the respective question. All analyses concerning adherence to medication were conducted with the subsample of N = 43 patients, who received medication.
