# Supplementary material for: Exploring potential influencing factors of inadherence to specialist aftercare and long-term medication in patients with acromegaly
Source: Pituitary. 2024 May 24;27(4):403–15. doi: 10.1007/s11102-024-01400-4 (PMC11289155; doi:10.1007/s11102-024-01400-4)
Supplement: Supplementary file 1 — Supplementary file1 (PDF 1365 KB) [file 11102_2024_1400_MOESM1_ESM.pdf]

Universitätsmedizin Essen
Universitätsklinikum
Klinik für Neurochirurgie
und Wirbelsäulenchirurgie

NB: Markings in red were added afterwards and indicate for which scales a mean score has been calculated.

Q1 - General questions about the person and life situation

Dear patient,

below you will find some questions about yourself and your life situation. Please answer all questions completely and do not omit any questions.

Thank you very much for your participation!

1. personal data

ID code (will be filled in by us)

Date

Age

Gender

- [ ] male
- [ ] diverse
- [ ] female

2. education

Highest school graduation:

- [ ] none
- [ ] Lower secondary school diploma
- [ ] Higher secondary school diploma
- [ ] Specialized high school diploma
- [ ] High school diploma

Do you have a completed apprenticeship?

- [ ] yes
- [ ] no

Do you have a university graduation?

- [ ] yes
- [ ] no

3. CURRENT living situation

I live:

- [ ] alone
- [ ] alone with children
- [ ] with my life partner
- [ ] with my life partner and children
- [ ] in a shared flat
- [ ] with my parents
- [ ] assisted

Marital status:

- [ ] single
- [ ] living in partnership
- [ ] married
- [ ] divorced
- [ ] widowed

4. physical data

Size (m)

Weight (kg)

Do you smoke? If yes, how much?

- [ ] Yes, ________________ cigarettes/day
- [ ] No

5. health insurance

How are you covered by health insurance?

- [ ] statutory health insurance
- [ ] private health insurance
  - [ ] without civil servant allowance
  - [ ] with civil servant allowance

6. CURRENT professional situation

Occupation

[ ]

In shift work?

- ☐ yes
- ☐ no

Professional situation

- ☐ Full-time
- ☐ Part-time
- ☐ unemployed
- ☐ Housewife / Househusband
- ☐ Occupational disability pension
- ☐ Early retirement
- ☐ Pension

6.1 If you are employed, are you currently on sick leave?

- ☐ Yes, since______/______/________(day/month/year)
- ☐ No

6.2 If you have not yet retired, have you applied for a pension?

- ☐ Yes
- ☐ No

6.3 Do you have a recognised degree of disability (e.g. by the pension office)?

☐ Yes   If yes, what is the degree? ________%

Has an upgrade been applied for?   ☐ Yes ☐ No

☐ No   If no, have you submitted a request?   ☐ Yes ☐ No

7. treatment of acromegaly

7.1 When were you diagnosed with acromegaly?

__________ /_________ (year/month if known)

7.2 Have you had surgery on your GH-producing pituitary adenoma due to acromegaly?

☐ Yes   ☐ No

7.3 If yes, how often and when?

1st OP   When?: _______ /_________ (year/month if known)

2nd OP   When?: _______ /_________ (year/month if known)

3rd OP   When?: _______ /_________ (year/month if known)

7.4 Have you ever received radiotherapy to the head?

☐ Yes   ☐ No   If yes, when? **from:** _______ /_______ (year/month if known)

until: _______ /________(year/month if known)

8. costs of medication

If you are currently taking medication, how high are the costs that you have to pay yourself through co-payments?

☐ about ____________________ Euro/year.

☐ I don't know.

9. general conditions of the treatment centre

How far is the centre treating your acromegaly from your home?

about _____________km

What are the approximate costs you incur for transport to the treating centre?

about _____________€ per appointment

10. household income

How many people in total live in your household?

_____________people

What is your monthly net household income?

☐ under 1000 €
☐ 1000 € to under 2000 €
☐ 2000 € to under 3000 €
☐ 3000 € to under 5000 €
☐ more than under 5000 €
☐ I don't know.

Universitätsmedizin Essen
Universitätsklinikum
Klinik für Neurochirurgie
und Wirbelsäulenchirurgie

Q2 -
Questionnaire on current disease status

Dear patient,

the following questions relate to your current acromegaly symptoms and your IGF-1 levels. Please answer all questions completely and do not omit any questions.

Thank you very much for your participation!

1. current symptoms

1.1 How much do you CURRENTLY suffer from the following symptoms?

	not at all	slightly	moderate	severe	very severe
Nausea	☐	☐	☐	☐	☐
Diabetes	☐	☐	☐	☐	☐
High blood pressure	☐	☐	☐	☐	☐
Depression	☐	☐	☐	☐	☐
Carpal tunnel syndrome	☐	☐	☐	☐	☐
Limited concentration	☐	☐	☐	☐	☐
Memory difficulties	☐	☐	☐	☐	☐
Daytime sleepiness	☐	☐	☐	☐	☐
Increased sweating	☐	☐	☐	☐	☐
Language change (sluggy language)	☐	☐	☐	☐	☐
Enlargement of hands/feet	☐	☐	☐	☐	☐
Coarsening of the facial features	☐	☐	☐	☐	☐
Widening of the interdental spaces	☐	☐	☐	☐	☐
Increased organ size (heart)	☐	☐	☐	☐	☐
Visual disturbances	☐	☐	☐	☐	☐
Joint pain	☐	☐	☐	☐	☐
Headache	☐	☐	☐	☐	☐
Swellings	☐	☐	☐	☐	☐

**For women:**	not at all	slightly	moderate	severe	very severe
Menstrual disorders	☐	☐	☐	☐	☐
Libido loss	☐	☐	☐	☐	☐
**For men**					
Libido loss	☐	☐	☐	☐	☐
Potency disorders	☐	☐	☐	☐	☐

Subjective symptomload

1.2 Do you <u>currently</u> have pain that you assume to be caused by acromegaly?

☐ Yes ☐ No

1.3 On how many days per month do you have pain on average?

about ____________ days / month

1.4 How severe is your pain on average?
Please mark the perceived pain intensity between 0 and 10 on the following scale:

0 |—|—|—|—|—|—|—|—|—|—| 10

2. IGF-1 values

2.1 How often is your IGF-1 level determined via a blood draw?

about every ___________ months

2.2 When was the last time your IGF-1 level was determined via a blood sample?

__________ /_________ (year/month if known)

2.3 What was your IGF-1 value at the last measurement? (if known)

- ☐ too high
- ☐ normal
- ☐ too low

Exact value (if known): ____________________

Universitätsmedizin Essen
Universitätsklinikum
Klinik für Neurochirurgie
und Wirbelsäulenchirurgie

Q3 -
Questionnaire on treatment adherence in acromegaly

Dear patient,

the following questions relate to the aftercare or drug therapy of your acromegaly disease. Please answer all questions completely and do not omit any questions.

Thank you very much for your participation!

1. Aftercare

1.1 How often do you go for endocrinological or neurosurgical follow-up?

☐ At least once a year, namely about _______ times/year.

☐ Less than once a year, namely about every ____________years.

☐ Not at all.

1.2 If you attend follow-up less than once a year, please give a reason (multiple answers possible):

	fully agree	rather agree	neither nor	rather not agree	not agree at all
I don't feel that it would be necessary.	☐	☐	☐	☐	☐
I have too little time.	☐	☐	☐	☐	☐
I don't feel like it.	☐	☐	☐	☐	☐
I don't like going to the doctor.	☐	☐	☐	☐	☐
I am afraid that I could be told that my illness has worsened.	☐	☐	☐	☐	☐
The effort of time and / or cost is too high for me.	☐	☐	☐	☐	☐

Other:

2. Medication

2.1 Have you EVER taken any of the following medication to treat acromegaly?

	Yes	No
Somatostatin analogues (e.g. lanreotide, octreotide, pasireotide)	☐	☐
Dopamine agonists (e.g. cabergoline, bromocriptine)	☐	☐
GH receptor antagonists (pegvisomant)	☐	☐

Other:

2.2 Have you been prescribed any medication to treat acromegaly that you should ACTUALLY take?

	Yes	No
Somatostatin analogues (e.g. lanreotide, octreotide, pasireotide)	☐	☐
Dopamine agonists (e.g. cabergoline, bromocriptine)	☐	☐
GH receptor antagonists (pegvisomant)	☐	☐

Other: ____________________

2.3 Do you take the medication prescribed for you?
Please only give details here for the medication that have CURRENTLY been prescribed for you (see question 2.2)!

	Yes	No
Somatostatin analogues (e.g. lanreotide, octreotide, pasireotide)	☐	☐
Dopamine agonists (e.g. cabergoline, bromocriptine)	☐	☐
GH receptor antagonists (pegvisomant)	☐	☐

Other: ____________________

2.4 What is your motivation for taking the medication prescribed to you?
(multiple answers possible)

	fully agree	rather agree	neither nor	rather not agree	not agree at all
I take them because my doctor advises me to.	☐	☐	☐	☐	☐
I take them because my physical performance has improved as a result.	☐	☐	☐	☐	☐
I take them because my cognitive performance has improved as a result.	☐	☐	☐	☐	☐
I take them because as a result I have less pain.	☐	☐	☐	☐	☐

Other: ____________________

2.5 Do you know the reason for the intake of your medication?

☐ Yes

☐ No

2.6 Do you know in <u>what dosage</u> you need to take of your medication?

☐ Yes

☐ No

2.7 Do you know <u>how often</u> you need to take your medication?

☐ Yes

☐ No

2.8 Do you always take your medication at the same time?
(Please tick only one answer)

☐ never ☐ rarely ☐ often ☐ always

2.9 Have you forgotten to take your medication in the last 4 weeks?
(Please tick only one answer)

☐ never ☐ rarely ☐ often ☐ always

2.10 Do you sometimes forget your medication at home when you are out?
(Please tick only one answer)

☐ never ☐ rarely ☐ often ☐ always

2.11 How important is it for you to take your medication regularly?
(Please tick only one answer)

☐ very important ☐ rather important ☐ rather unimportant ☐ unimportant

2.12 Do you sometimes intentionally skip taking a medication?
(Please tick only one answer)

☐ never ☐ rarely ☐ often ☐ always

2.13 Do you sometimes reduce the dosage of your medication?
(Please tick only one answer)

☐ never ☐ rarely ☐ often ☐ always

Adherence score

2.14 If you occasionally skip taking your medication or reduce the dosage, please state the reasons.

Universitätsmedizin Essen
Universitätsklinikum
Klinik für Neurochirurgie
und Wirbelsäulenchirurgie

Q4 - Questionnaire on other diseases and complaints

Dear patient,

the following questions are about diseases that may coexist with your acromegaly disease. Please answer all questions completely and do not omit any questions.

Thank you very much for your participation!

1. Are there any other diseases or physical complaints in addition to acromegaly?

☐ Yes ☐ No *(continue with the next questionnaire)*

If yes, what secondary disease(s) and their effects do you have?

2. Is there a change in the sugar metabolism? ☐ Yes ☐ No *(continue with question 3)*

2.1 If yes, is there an insulin requirement? ☐ Yes ☐ No

2.2 If **not** insulin dependent, are you taking any other antidiabetic medication? ☐ Yes ☐ No

3. Do you have any of the following vascular or cardiac diseases? *(Multiple answers possible)*

☐ High blood pressure ☐ Heart failure ☐ Heart valve defect

☐ Cardiac arrhythmia

☐ Other:

4. Do you have a lung or respiratory disease?

☐ Yes ☐ No *(continue with question 5)*

4.1 If yes, have you been diagnosed with sleep apnoea syndrome? ☐ Yes ☐ No

4.2 If yes, do you use a CPAP machine regularly? ☐ Yes ☐ No

4.3 What other disease(s) of the lungs or respiratory tract do you have?

5. Do you have any musculoskeletal disorders (muscles, bones, tendons, ligaments, joints)?

☐ Yes ☐ No *(continue with question 6)*

5.1 If yes, which disease(s) / complaints do you have?

6. Do you currently have or have you had a malignant disease (cancer) in the past?

☐ Yes ☐ No *(continue with question 7)*

6.1 Is the disease current or was it in the past?

☐ current ☐ The disease is no longer current, I am in remission.

6.2 When was the diagnosis made?

__________ /_________ (year/month if known)

6.3 Which malignant disease do you have?

7. Do you have a limitation of the field of vision?

☐ Yes ☐ No

8. Do you regularly suffer from headaches?

☐ Yes ☐ No *(continue with question 9)*

8.1 If yes, how many headache days do you have on average per month?

about __________ days / month

9. What medication do you take for your present symptoms (excluding medication you are taking as part of the acromegaly treatment)?

Medication	Dosage	Since when (month/ year)

Universitätsmedizin Essen
Universitätsklinikum
Klinik für Neurochirurgie
und Wirbelsäulenchirurgie

Q5 -
Questionnaire about the therapy of acromegaly

Dear Patient,

the following questions deal with the therapy of acromegaly and the perceived effects of the therapy on your physical and mental condition. Please answer all questions completely and do not omit any questions.

Thank you very much for your participation.

1. How long have you been under medical treatment for your acromegaly disease?

__________/_________ (year/month if known)

2. Did you interrupt the therapy at any time?

☐ Yes ☐ No *(continue with question 3)*

2.1 If yes, how long did you interrupt the therapy?

☐ 1 year or shorter ☐ 1-5 years ☐ longer than 5 years

2.2 If yes, what was the reason for the therapy interruption?

	fully agree	rather agree	neither nor	rather not agree	not agree at all
Lack of motivation	☐	☐	☐	☐	☐
Dissatisfaction with the physician	☐	☐	☐	☐	☐
Organisational problem	☐	☐	☐	☐	☐
Improvement of health condition, so that the therapy was not felt to be necessary	☐	☐	☐	☐	☐
Deterioration of health so that it was not possible to attend appointments	☐	☐	☐	☐	☐
Dissatisfaction with the success of the therapy	☐	☐	☐	☐	☐

☐ Other:

3. Overall, how successful do you experience your therapy?

very successful	successful	moderate	of little success	not successful at all
☐	☐	☐	☐	☐

4. Has your physical or mental (psychological) condition changed since you started therapy?

	much better	better	unchanged	worsened	strongly worsened
My physical condition since I started therapy has been ...	☐	☐	☐	☐	☐
My mental (psychological) condition since I started therapy has been ...	☐	☐	☐	☐	☐

5. What specific changes have you noticed since you started therapy?

	fully agree	rather agree	neither nor	rather not agree	not agree at all
I can accept the physical changes caused by acromegaly.	☐	☐	☐	☐	☐
I am more open about my disease.	☐	☐	☐	☐	☐
I can manage my everyday life better again.	☐	☐	☐	☐	☐
I understand the disease and the changes it brings or has brought.	☐	☐	☐	☐	☐

Perceived beneficial effects

	fully agree	rather agree	neither nor	rather not agree	not agree at all
I feel stressed by the doctor's appointments.	☐	☐	☐	☐	☐
Therapy takes up a lot of space in my everyday life.	☐	☐	☐	☐	☐
I am burdened by taking the medication every day.	☐	☐	☐	☐	☐
I suffer from the side effects of the medication.	☐	☐	☐	☐	☐

Perceived adverse effects

☐ Other:

Universitätsmedizin Essen
Universitätsklinikum
Klinik für Neurochirurgie
und Wirbelsäulenchirurgie

Q6 - Questionnaire on doctor-patient communication

Dear patient,

the following questions are about the communication between you and your doctor in the context of treating your acromegaly. When answering the questions, please refer to the doctor you talk to most about your acromegaly. Please answer all questions completely and do not omit any questions.

Thank you very much for your participation.

1. **How well were you informed by your doctor about the following aspects related to your acromegaly disease?**

	very good	good	moderate	bad	very bad
Development of the disease	☐	☐	☐	☐	☐
Course of the disease	☐	☐	☐	☐	☐
Necessity of regular follow-up examinations	☐	☐	☐	☐	☐
Possible effects on everyday life	☐	☐	☐	☐	☐
Reasons for taking medication	☐	☐	☐	☐	☐
Possibilities to obtain further reliable information (literature, information brochures etc.)	☐	☐	☐	☐	☐
Opportunities to participate in self-help groups	☐	☐	☐	☐	☐
Side effects of the medication	☐	☐	☐	☐	☐
Therapy options	☐	☐	☐	☐	☐

Education on acromegaly

2. How would you assess the information and how it is communicated to you by your doctor?

	fully agree	rather agree	neither nor	rather not agree	not agree at all
I have the feeling that my doctor takes enough time for me.	☐	☐	☐	☐	☐
I always feel I can ask questions if I don't understand something.	☐	☐	☐	☐	☐
The amount of information is just right to understand everything.	☐	☐	☐	☐	☐
After the diagnosis, I was emotionally stable enough to understand the information.	☐	☐	☐	☐	☐

Quality of information provision

	fully agree	rather agree	neither nor	rather not agree	not agree at all
The information is explained in simple, easy-to-understand terms.	☐	☐	☐	☐	☐
My doctor asks if I am getting enough information.	☐	☐	☐	☐	☐
I have the feeling that my doctor assumes that I already have enough information and therefore explains too little.	☐	☐	☐	☐	☐
I have understood the information.	☐	☐	☐	☐	☐
I feel sufficiently informed by the information.	☐	☐	☐	☐	☐
My doctor uses specialist terminology to an appropriate and understandable extent.	☐	☐	☐	☐	☐

Quality of information provision

3. How did you obtain further information outside the conversation with your doctor?

☐ Internet ☐ Networking with other affected people ☐ No further information obtained at all

☐ Information brochure ☐ (Text)books

☐ Other:

4. How long does it usually take to talk to your doctor at a routine appointment?

☐ less than 5 minutes ☐ between 5 and 10 minutes

☐ between 10 and 20 minutes ☐ more than 20 minutes

4.1 Do you feel that this duration is appropriate?

far too long	a little too long	just right	a little too short	far too short
☐	☐	☐	☐	☐

5. How well did your doctor discuss the following aspects of a potentially expected therapy success with you before starting the therapy?

	very good	good	moderate	bad	very bad
Improvement of hormone levels	☐	☐	☐	☐	☐
Improvement of physical complaints	☐	☐	☐	☐	☐
Reduction of psychological complaints	☐	☐	☐	☐	☐
Reduction of restrictions in everyday life	☐	☐	☐	☐	☐

6) How well does your doctor discuss the actual course of therapy with you during the therapy?

very good	good	moderate	bad	very bad
☐	☐	☐	☐	☐

7. How well did your doctor explain to you that you will need permanent follow-up care (follow-up appointments and, if necessary, medication) after the therapy has taken place?

very good	good	moderate	bad	very bad
☐	☐	☐	☐	☐

8. How well did your doctor explain to you the reasons why it is so important that you follow the recommendations for treatment that have been agreed upon (attend follow-up appointments, take medication according to plan)?

very good	good	moderate	bad	very bad
☐	☐	☐	☐	☐

9. How well does your doctor deal with possible difficulties in implementing the treatment recommendations (follow-up appointments and taking medication)?

very good	good	moderate	bad	very bad
☐	☐	☐	☐	☐

Universitätsmedizin Essen
Universitätsklinikum
Klinik für Neurochirurgie
und Wirbelsäulenchirurgie

Q7 - Questionnaire on the perceived therapy needs

Dear patient,

the following questions deal with the personal assessment of your need for therapy as well as your expectations and wishes with regard to therapy. Please answer all questions completely and do not omit any questions.

Thank you very much for your participation.

1. What is your own estimation of the need for medical therapy in regard to your acromegaly disease?

☐ no need ☐ low demand ☐ average demand ☐ high demand ☐ very high demand

1.1 Please give reasons for your assessment.

	fully agree	rather agree	neither nor	rather not agree	not agree at all
My physical condition is good at the moment.	☐	☐	☐	☐	☐
My mental state is good at the moment.	☐	☐	☐	☐	☐
Others are worse off than me and need treatment more urgently.	☐	☐	☐	☐	☐
The therapy doesn't bring any change anyway, so I might as well not do it.	☐	☐	☐	☐	☐

☐ Other:

2a. What are your expectations of the medical treatment of your acromegaly disease?

	fully agree	rather agree	neither nor	rather not agree	not agree at all
I expect the treatment to improve my performance in everyday life.	☐	☐	☐	☐	☐
I expect the treatment to alleviate my physical discomfort.	☐	☐	☐	☐	☐
I expect the treatment to improve my psychological well-being.	☐	☐	☐	☐	☐
I expect a quick success of the therapy.	☐	☐	☐	☐	☐
I expect that the medication will prevent the need for pituitary surgery.	☐	☐	☐	☐	☐
I expect to have to take less medication in the future as a result of the treatment.	☐	☐	☐	☐	☐

Positive treatment expectations

	fully agree	rather agree	neither nor	rather not agree	not agree at all
I expect to have fewer doctor's appointments in the future as a result of the treatment.	☐	☐	☐	☐	☐

Positive treatment expectations

☐ Other expectations:

2b. What wishes do you have for the medical treatment of your acromegaly disease?

	fully agree	rather agree	neither nor	rather not agree	not agree at all
I want to be treated by an expert.	☐	☐	☐	☐	☐
I want to have a constant doctor to refer to and not be treated by constantly changing doctors.	☐	☐	☐	☐	☐
I would like to be fully informed about the treatment and treatment options.	☐	☐	☐	☐	☐
I want to be involved in the decision about the therapy.	☐	☐	☐	☐	☐
I would like the doctor to have enough time and patience to answer my questions.	☐	☐	☐	☐	☐

☐ Other wishes:

2c. What concerns do you have about the medical treatment of your acromegaly disease?

	fully agree	rather agree	neither nor	rather not agree	not agree at all
I am worried that the treatment will not work.	☐	☐	☐	☐	☐
I am worried that the treatment will not bring the improvement I hope for.	☐	☐	☐	☐	☐
I am worried that the treatment will make my symptoms and limitations worse.	☐	☐	☐	☐	☐
I am worried that I will get unpleasant side effects from the medication.	☐	☐	☐	☐	☐
I am worried that the treatment will take up a lot of time and determine my everyday life.	☐	☐	☐	☐	☐

Negative treatment expectations

☐ Other concerns:

3. How do you currently rate the extent of your acromegaly disease?

	fully agree	rather agree	neither nor	rather not agree	not agree at all
I have a serious illness.	☐	☐	☐	☐	☐
I attribute my physical impairments to my acromegaly disease.	☐	☐	☐	☐	☐
I attribute my mental impairments to my acromegaly disease.	☐	☐	☐	☐	☐
My acromegaly disease causes clearly noticeable symptoms for me.	☐	☐	☐	☐	☐
My acromegaly disease is a big burden for me.	☐	☐	☐	☐	☐
